# Omega-3 polyunsatured fatty acids and physical performance across the lifespan: a narrative review

**DOI:** 10.3389/fnut.2024.1414132

**Published:** 2024-06-20

**Authors:** Domenico Azzolino, Camilla Bertoni, Valentina De Cosmi, Giulia Carla Immacolata Spolidoro, Carlo Agostoni, Tiziano Lucchi, Alessandra Mazzocchi

**Affiliations:** ^1^Geriatric Unit, Fondazione IRCCS Ca’ Granda Ospedale Maggiore Policlinico di Milano, Milan, Italy; ^2^Department of Veterinary Sciences for Health, Animal Production and Food Safety, University of Milan, Milan, Italy; ^3^Department of Food Safety, Nutrition and Veterinary Public Health, Istituto Superiore di Sanità—Italian National Institute of Health, Rome, Italy; ^4^Department of Clinical and Community Sciences, University of Milan, Milan, Italy; ^5^Pediatric Intermediate Care Unit, Fondazione IRCCS Ca’ Granda Ospedale Maggiore Policlinico, Milan, Italy

**Keywords:** aging, nutrition, life course, sarcopenia, omega-3 PUFAs, frailty

## Abstract

**Background and Aims:**

Physical performance is a major contributor of mobility and independence during older life. Despite a progressive decline in musculoskeletal function starts from middle age, several factors acting during the life-course can negatively influence musculoskeletal functional capacities. Lifestyle interventions incorporating nutrition and physical exercise can help maximizing the muscle functional capacities in early life as well as preserving them later in life. Among various dietary compounds, omega-3 polyunsaturated fatty acids (PUFAs) are gaining growing attention for their potential effects on muscle membrane composition and muscle function. Indeed, several pathways are enhanced, such as an attenuation of pro-inflammatory oxidative stress, mitochondrial function, activation of the mammalian target of rapamycin (mTOR) signaling and reduction of insulin resistance.

**Methods:**

We performed a narrative review to explore the existing literature on the relationship between omega-3 PUFAs and physical performance across the life-course.

**Results:**

Growing evidence from randomized controlled trials (RCTs) suggests beneficial effects of omega-3 PUFAs on muscle function, including physical performance parameters in mid to later life. On the other hand, despite a direct association in early life is not available in literature, some mechanisms by which omega-3 PUFAs may contribute to improved adult physical performance could be hypothesized.

**Conclusion:**

Omega-3 PUFAs are gaining growing attention for their positive effect on muscle function parameters. The integration of physical function measures in future studies would be of great interest to explore whether omega-3 PUFAs could contribute to improved muscle function, starting from early life and extending throughout the lifespan. However, larger and high-quality RCTs are needed to fully elucidate the beneficial effects of omega-3 PUFAs supplementation on muscle mass and function.

## Introduction

Advancing age is characterized by a progressive and generalized decline in muscle mass and function, the so-called “sarcopenia.” However, sarcopenia can occur earlier in life (1, 2). The original definition of sarcopenia focused on muscle mass as stand-alone. Subsequently, much more emphasis has been given to muscle function such that it currently comes to the forefront of international guidelines (3, 4). Physical performance, a major contributor of mobility and independence during older life (5), has been defined as “an objectively measured whole-body function related to locomotion” (3, 6). The multidimensional concept of physical performance is not merely limited to skeletal muscle but also involves central and peripheral nervous function including balance (3, 6). Low physical performance has been formerly considered a core component of sarcopenia (3, 7) and has been widely associated with adverse outcomes including frailty, disability and subsequent death (3, 8–14). Consequently, low physical performance is used to identify the severity of sarcopenia (3). There is a large consensus on the key role that physical function, and in particular mobility, plays in the determination of frailty status (15, 16), regardless of the operational definition used (17). At the same time, the relationship between frailty and physical performance may be bi-directional since frailty status can also negatively affect mobility and physical function. In fact, physical performance measures are often used as an outcome measure in most of the trials targeting frailty and sarcopenia. Frailty and sarcopenia can be considered complementary in many aspects. To date, the physical frailty phenotype proposed by Fried et al. (18) shows a remarkable overlap with sarcopenia. The Physical Frailty and Sarcopenia (PF&S) model (15) has been therefore suggested as a possible solution to combine the two entities (i.e., frailty and sarcopenia) into a unique operational definition. Furthermore, the large body of literature about physical function impairment as well as the presence of dedicated measures that are widely accepted (e.g., short physical performance battery, handgrip strength, gait speed), make the PF&S model an easy-to-implement model to capture both frailty and sarcopenia (15). It is indeed largely agreed that the physical function impairment that results from the combination of frailty and sarcopenia acquires completely different connotations toward worst outcomes (19).

**Figure 1:**
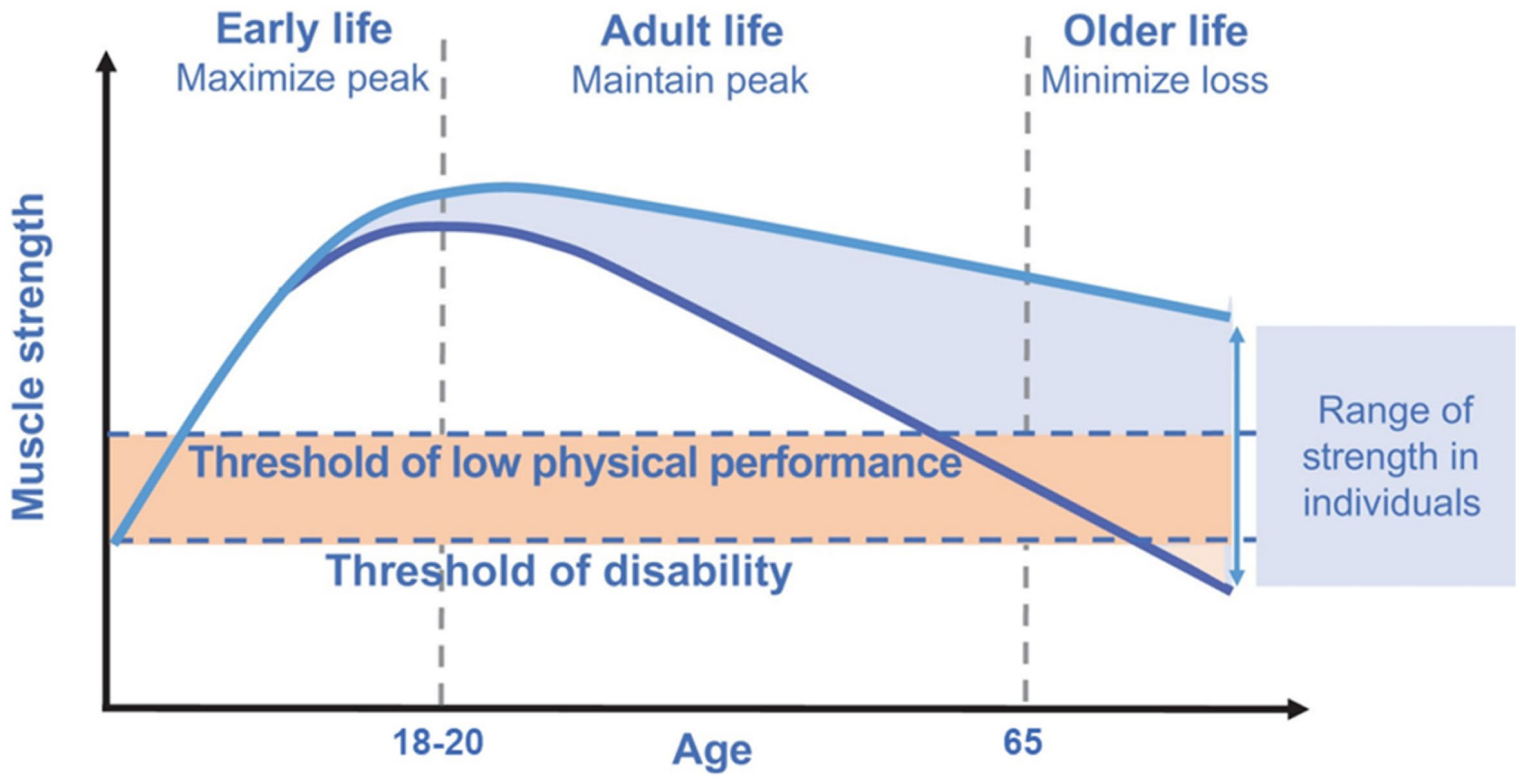
Muscle strength across the life course. Modified from Cruz-Jentoft et al. (3), licensed under CC BY-NC 4.0. The rate of decline in muscle strength is reflected by the peak attained during early life. Maximizing the peak of muscle strength in early life as well as maintaining this peak during adult life is pivotal to minimize losses during older life.

Despite a progressive decline in musculoskeletal function starts from middle age, several factors acting during the life course can influence musculoskeletal functional capacities. Starting from early life, each individual rapidly acquires supporting muscle functions to reach a peak or a plateau nearly at the end of adolescence period. Subsequently, after the fourth decade of life, a progressive decline in muscle mass (i.e., ~1–2% per year) and strength (i.e., ~1.5% per year) is seen (20). Kaymak et al. (3), in a comment to the European Working Group on Sarcopenia in Older People revised consensus (EWGSOP2), suggested that measurement of muscle power (intended as the product of strength and velocity) is more relevant than muscle strength as stand-alone in reflecting physical performance. Accordingly, muscle power has been suggested as the most relevant measure of muscle function, being more strongly correlated with functional performance than strength as stand-alone in older people. An example may be the chair stand test that requires both strength and velocity for its execution (3). In the early phases of muscle decline (i.e., initial decrease in muscle mass and strength), an individual could still have a preserved physical performance and may be very far from the threshold of disability (3) (Figure 1). Hall et al. (5), in the Physical Performance Across the Life-span Study, reported that physical performance is almost stable in the first two decades of adulthood (i.e., from 30 to 50 years of age) with a progressive decline in the middle years (i.e., 50+) and late adulthood. Besides genetic and lifestyle factors operating across the life course, also some pathological conditions can accelerate this degenerative process (with a consequent progression toward functional impairment and disability). Lifestyle interventions incorporating nutrition and physical exercise are able to slow or reverse this process (Figure 1) (3, 21). The rate of decline in muscle mass and function is also reflected by their peaks attained during early life (2). Indeed, it is essential to maximize muscle function in early life as well as maintain this peak during adult life to minimize losses during older life (3).

Nutritional strategies to counteract muscle mass and function decline are mainly based on protein supplementation combined with adequate calorie intake while there is limited knowledge on other nutritional interventions (22). Furthermore, the efficacy of nutritional interventions is enhanced when combined with physical activity (i.e., resistance training) (23). Inflammation and oxidative stress are considered hallmarks of the aging process (24) influencing the rate of functional decline observed in aging. Therefore, supplementation with individual nutrients sharing antioxidant and anti-inflammatory properties has recently gained attention for potential effects against the age-related functional decline (1, 25, 26). Within the plethora of various dietary supplements, polyunsaturated fatty acids (PUFAs), particularly omega-3 PUFAs and derived long-chain PUFAs (LC-PUFAs), are of particular interest for their potential effects on muscle function and thus on physical performance through various mechanisms. Omega-3 PUFAs may promote muscle anabolism through activation of the mammalian target of rapamycin (mTOR) signaling and reduction of insulin resistance (27, 28). Furthermore, omega-3 PUFAs are widely acknowledged as nutrients with clear anti-inflammatory and antioxidant properties (27, 29, 30).

This narrative review aims to provide an overview of current knowledge about the role of omega-3 PUFAs on physical performance across the lifespan.

### The role of PUFAs in the body

**Figure 2:**
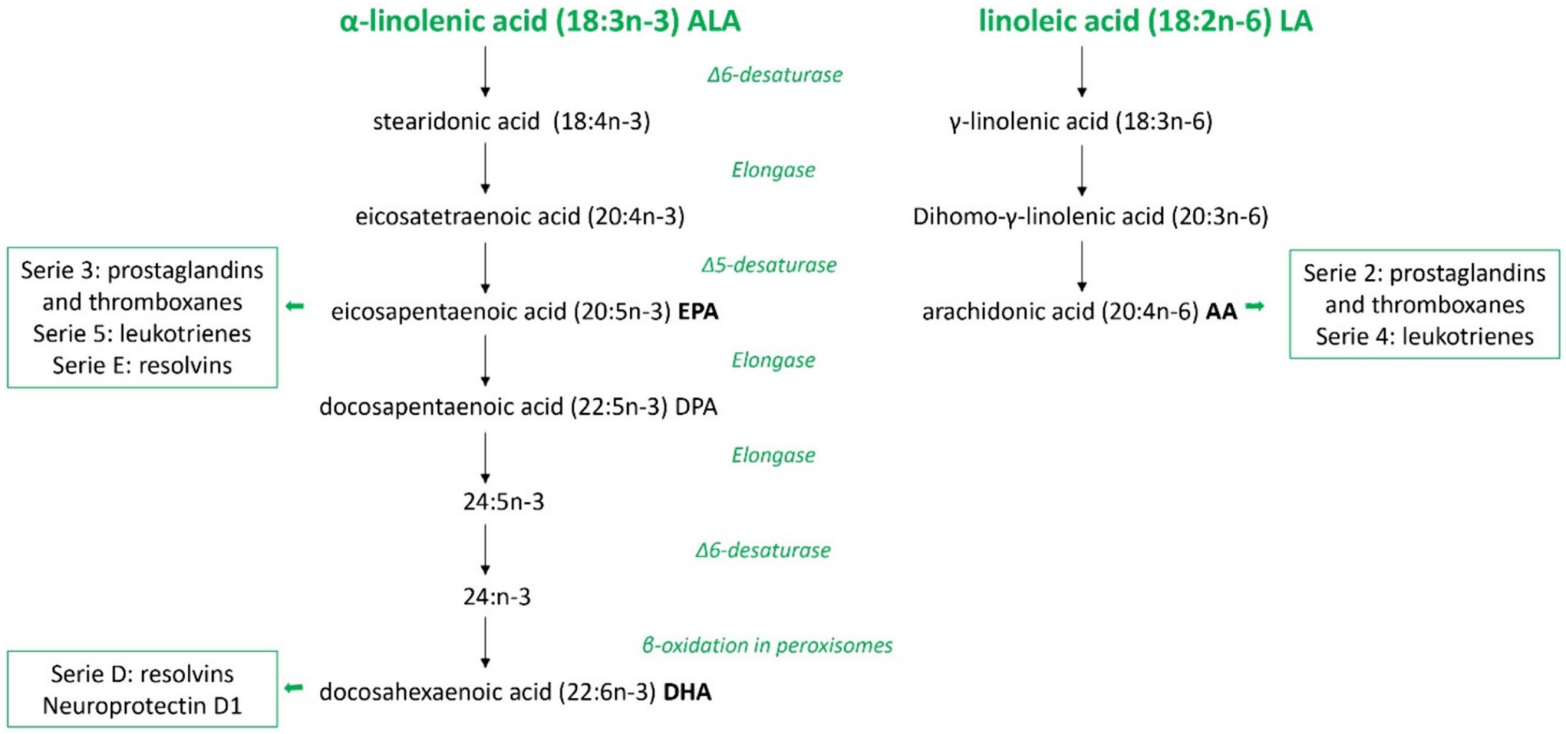
Overview of polyunsaturated fatty acids metabolism in mammals. Schematic metabolic pathways of the two families of polyunsaturated fatty acids in mammals, omega-3 and omega-6, from the precursors α-linolenic acid and linoleic acid, respectively. In this figure are represented all the consecutive desaturation and elongation steps and the names of major eicosanoids derived. ALA, α-linolenic acid; LA, linoleic acid; EPA, eicosapentaenoic acid; DPA, docosapentaenoic acid; DHA, docosahexaenoic acid; AA, arachidonic acid.

Linoleic acid (LA, 18:2) and α-linolenic acid (ALA, 18:3) are considered essential fatty acids (EFAs) because of the absence of enzymes necessary for their production in humans and other mammals. Instead, they are obtained from plants or other organisms that possess enzymatic pathways for their synthesis. They must be part of the diet and, once introduced into the body, they can be further metabolized in the liver by the enzymes Δ6 and Δ5 desaturases and elongases to generate PUFAs (31) (see Figure 2).

LA is the precursor of the omega-6 PUFA family and arachidonic acid (AA) is its derived compound, while ALA, eicosapentaenoic acid (EPA), and docosahexaenoic acid (DHA) belong to the omega-3 PUFA family. These two groups of fatty acids influence in different ways the property of the cell membranes in terms of fluidity and biological effects. DHA, EPA, and AA compete for the sn-2 position on membrane phospholipids. Therefore, their relative abundance in the membrane influences their availability as substrates for the same metabolic pathway enzymes, such as cyclooxygenases and lipoxygenases. Consequently, this balance affects the production of bioactive compounds with antagonistic roles involved in various disease processes (32). Generally, AA-derived metabolites have pro-inflammatory properties, whereas EPA-derived compounds are less inflammatory. DHA-derivates have anti-inflammatory and pro-resolution activities contributing to speeding up the inflammatory response’s physiological resolution (33, 34).

Omega-3 PUFAs such as ALA and DHA and omega-6 PUFAs, such as LA and AA, are important structural components of cell membranes mainly within phospholipids. DHA is necessary for the development of brain functions and retinal functions associated to vision (35, 36). The incorporation of this omega-3 metabolite takes place at uniquely high levels in the central nervous system, where omega-3 PUFA are main determinant of membrane PUFA composition and unsaturation (37). Once high levels of DHA are established in the brain, they tend to be sustained throughout later life. This maintenance likely relies on an optimal dietary supply, particularly considering the potential decrease in efficiency of precursor conversion by the enzymatic pathway among older individuals (35, 38).

The membrane PUFA composition seems to be more responsive to dietary DHA compared to intake of LA and AA, showing a high sensitivity to dietary variations in PUFA-supply (39).

DHA, derived from ALA, has relevant metabolic activities. Besides hypolipidemic properties, reducing blood concentrations of triglycerides, DHA contributes to protect the central nervous system from reactive oxygen species, together with antioxidant properties, capable of shutting down the upstream inflammatory cascade. Furthermore, DHA exhibits immunomodulatory and antiallergic activities, contributing to its overall neuroprotective effects. From intrauterine life through later ages, DHA contributes to the maintenance of cognitive abilities and the prevention of neuropsychiatric and neurodegenerative disorders (40).

DHA and EPA share anti-inflammatory and inflammation-resolving properties including the partial inhibition of leucocyte chemotaxis, adhesion molecule expression and leukocyte-endothelial adhesive interactions, production of eicosanoids such as prostaglandins and leukotrienes from the AA, as well as the production of pro-inflammatory cytokines (30). These anti-inflammatory and pro-resolving effects show to be relevant to improve clinical outcomes in different therapeutic areas, supporting the protective role of the immune system (41). Omega-3 PUFAs seem to be involved in the activation of cells from both the innate and the adaptive immune system (42). In particular, in the innate immune cells omega-3 PUFAs (1) reduce neutrophil migration and increase their phagocytosis, (2) reduce pro-inflammatory cytokine release, increase phagocytosis and M2 macrophages phenotype that promote tissue repair at macrophage level and (3) reduce presentation at dendritic cells level (42). In the adaptive immune cells, omega-3 PUFAs limit excessive B-cells responses, increase T regulatory cells differentiation and function while reduce T helper 17 differentiation, and limit the release of pro-inflammatory cytokines (42, 43). Consistent with aims of the present review, in older populations growing evidence suggests a role of omega-3 PUFAs in the maintenance of muscle mass and function and in the musculoskeletal health in general (44).

### Main mechanisms of omega-3 PUFAs in the muscle

**Figure 3:**
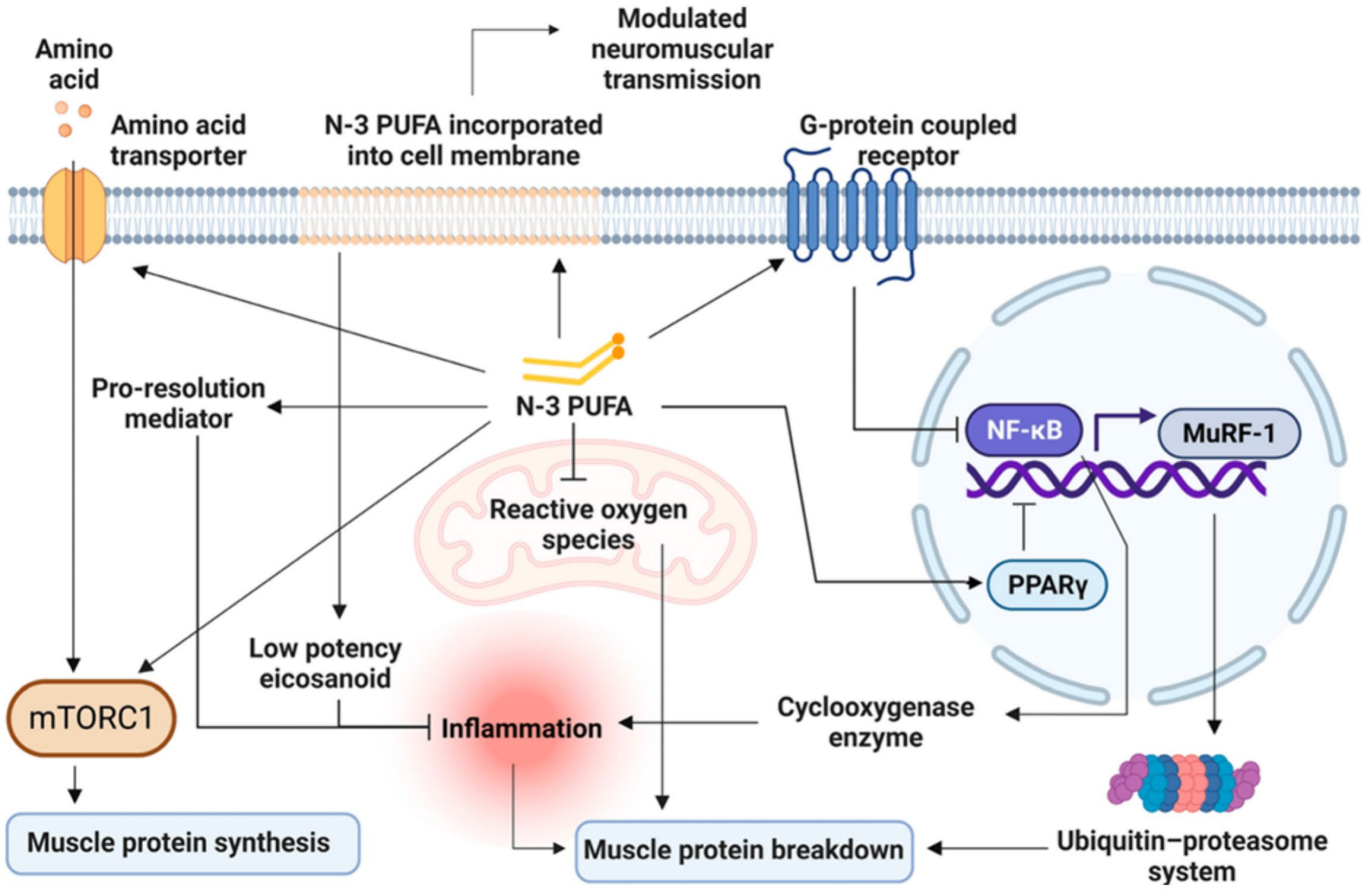
Overview of the main mechanisms by which Omega-3 PUFAs can influence muscle parameters. Modified from Therdyothin et al. (47), licensed under CC BY 4.0. Omega-3 PUFAs, incorporated into the cell membrane, modulate neuromuscular transmission and seem to directly stimulate mTORC1, both as stand-alone and synergically with amino acids ingestion thus enhancing muscle protein synthesis. Omega-3 PUFAs also counteract inflammatory processes through less production of inflammatory mediators as well as by the release of pro-resolution mediators and by reducing ROS production at the mitochondrial level, thus decreasing muscle protein breakdown. Omega-3 PUFAs also act as ligands for G-protein coupled receptors (GPCRs) and induce the activation of the peroxisome proliferator-activated receptors (PPARs), with the consequent inhibition of nuclear factor kappa B (NFκB). In turn, the inhibition of NFκB leads to a reduced cyclooxygenase production resulting in a decreased inflammatory response finally reducing muscle protein breakdown. The inhibition of NF-κB also leads to the downregulation of the muscle ring finger-1 (MuRF-1) gene counteracting the ubiquitin-proteasome system and thus reducing muscle protein breakdown. n-3 PUFA, omega-3 polyunsaturated fatty acid; mTORC1, mammalian target of rapamycin complex; NFκB, nuclear factor kappa B; MuRF-1, muscle ring finger-1; PPARs, peroxisome proliferator-activated receptors.

Most studies on omega-3 PUFAs have been primarily focused on cellular and molecular mechanisms underlying muscle protein metabolism (45). Accordingly, the main mechanisms through which omega-3 PUFAs could benefit muscle parameters seem to be (1) both anti-catabolic and anabolic effects on muscle protein synthesis (2) modulation of insulin sensitivity (3) amelioration of mitochondrial functioning, inflammation and muscle fiber contractile properties (4) neuroprotective and motor neuron excitability properties (46). Figure 3 presents an overview on the main mechanisms, which will be discussed in detail by each life stage (e.g., early life, adult and older life) in the following specific sections, by which omega-3 PUFAs can influence muscle parameters.

Omega-3 PUFAs EPA and DHA, both as stand-alone and synergically with amino acids ingestion, seem to directly stimulate mTORC1 thus enhancing muscle protein synthesis (Figure 3). However, it should be considered that while the acute activation of mTORC1 could promote muscle protein synthesis (48), the prolonged activation of mTORC1 has been associated with severe muscle atrophy, mainly because of decreased autophagy in the muscle (49). Therefore, it seems that alternating periods of high and low mTORC1 activation, as occurring with a healthy diet incorporating omega-3 PUFAs, is the key for an optimal muscle function (50). Omega-3 PUFAs seem also to improve mitochondrial function, mainly by reducing non-mitochondrial respiration and by augmenting the reserve respiratory capacity and bioenergetics (51). By reducing ROS production at the mitochondrial level, omega-3 PUFAs seem also to reduce muscle protein breakdown (47). Additionally, omega-3 PUFAs seem to counteract insulin resistance by improving mitochondrial function and bioenergetics as well as by modulating phospholipid membranes (52, 53), but also by increasing serum levels of insulin-like growth factor 1 (IGF-1). In turn, IGF-1 stimulates muscle protein synthesis via mTORC1-dependent and independent pathways (47, 54).

Omega-3 PUFAs could inhibit muscle protein breakdown also by acting as ligands for G-protein coupled receptors (GPCRs) and by activating the peroxisome proliferator-activated receptors (PPARs), with the consequent inhibition of nuclear factor kappa B (NFκB). In turn, the inhibition of NFκB leads to a reduced cyclooxygenase production resulting in a decreased inflammatory response (55) (Figure 3). The inhibition of NF-κB also leads to the downregulation of the muscle ring finger-1 (MuRF-1) gene counteracting the ubiquitin-proteasome system and thus reducing muscle protein breakdown (47). Furthermore, PPARs which are transcription factors activated by fatty acids and their derivatives, are involved in development, metabolism, inflammation, and many cellular processes in different tissues including the muscle (56). In particular, there are three different PPAR isotypes: (1) PPARα is highly expressed in tissues, like skeletal muscle, with effective fatty acid catabolism; (2) PPARβ/δ, which is more ubiquitously with a predominance in the skeletal muscle, is implicated in energy metabolism, mitochondrial biogenesis, and fiber-type switching; (3) PPARγ is highly expressed in adipocytes, but it is also involved in fat deposition in the muscle (56). Indeed, beyond NF-κB inhibition, PPARγ activation seems to play a relevant role in the inhibition of myosteatosis (i.e., intramuscular and intermuscular fat infiltration) and muscle fiber type switching (56) which are key features in the age-related sarcopenia (1). In particular, fat deposition in the muscle with its lipotoxic action, can exert detrimental effects on both muscle quality and strength, also negatively affecting mobility function (57–59). These effects are even more magnified when sarcopenia is accompanied by obesity (60). Additionally, PPARγ is a key regulator of glucose homeostasis and insulin sensitivity in the human skeletal muscle (61), with abnormalities in its expression being involved in skeletal muscle insulin resistance, especially in the presence of obesity and/or type II diabetes (62). The effects of omega-3 PUFAs supplementation on PPARγ activity have been demonstrated also in young athletes (e.g., age range of 20 to 30 years) who were supplemented with 2000 mg/day of omega-3 PUFAs (EPA: 360 mg, DHA: 240 mg) or placebo (2000 mg/day edible paraffin) for 3 weeks (63). The authors found that omega-3 PUFAs supplementation was significantly associated with the up-regulation of PPARγ, with an increase in resting energy expenditure and appetite (63). Also a recent meta-analysis of randomized controlled trials (RCTs) (64), involving both young, middle-aged and older people, showed that omega-3 PUFAs supplementation at varying doses (from 2000 mg/day to 7,000 mg/day) and with varying duration (from 3 to 48 weeks) led to a significant up-regulation of PPAR-γ gene expression. Finally, the incorporation of omega-3 PUFAs in the phospholipid bilayers of the cell membrane favors membrane fluidity and modulates neuromuscular transmission (Figure 3) resulting in greater muscle strength (47, 65–67).

### Physical performance measures

The assessment of physical performance in older people can be envisioned as a summary marker of functional status as well as of the underlying biology of ageing (8). Tests of physical performance are strongly associated with frailty, disability and death in older people (9–14) and are used to identify the severity of sarcopenia (3). Physical performance measures are thus intended to monitor the evolution of functional status over time or the change after an intervention (68, 69), so such tests are often used as an outcome measure in most trials (3). Much research has been conducted in large, prospective studies of older populations, assessing physical performance in several ways. The main tests of physical performance in older people are summarized in Table 1. Physical performance is usually measured in older people by the Short Physical Performance Battery (SPPB) (11, 72), the 400-meter walk test (400-MWT) (74), gait speed (70, 71), and the Timed-Up and Go (TUG) test (73), as suggested by the EWGSOP2 (3). Shortly, the SPPB test combines the assessment of gait speed, a balance test, and a chair stand test. The SPPB scores range from 0 to 12, with a score of ≤8 points indicating poor physical performance (11, 72). However, the SPPB is frequently used in research rather than in clinical practice because of its length of administration (i.e., at least 10 min) (3). The 400-MWT evaluates both walking ability and endurance and consists of completing 20 laps of 20 m as fast as possible, with up to two rest stops during the test that are allowed. Non-completion or ≥6 min for completion of this test indicates poor physical performance (74). Also in this case, the length of the 400-MWT as well as the need for a corridor at least 20 m long make it difficult to implement in routine clinical practice. However, in the geriatric context, some tests with shorter distances (e.g., 4, 7, and 10 Meter Walk Test) have been proposed as good alternatives showing high test–retest reliability and validity in measuring walking speed (76–78). However, also the 10-meter walk test (10-MWT) requires a corridor 20 m long making it difficult to implement in most clinical settings (79). Indeed, the 4-meter walk test (4-MWT), which is part of the SPPB test, is considered a valid alternative to the 10-MWT in both clinical and research settings (3, 80), with a cut-off ≤0.8 m/s that has been advised to indicate poor physical performance (i.e., severe sarcopenia) (3). Gait speed is instead considered an easy-to-implement highly reliable measure and it is advised, for its convenience, by EWGSOP2 for physical performance assessment (3). Likewise, the TUG test is widely used in the geriatric context for its simplicity and reliability. In particular, the TUG test asks the participant to rise from a chair, walk up to a marker 3 m away, turn around, walk back to the chair and sit down again, with a cut-off of ≥20 s suggested as indicative of poor physical performance (3, 73). To better capture the age-related modifications in functional status, physical performance should be assessed before reaching old age, taking into consideration the determinants that influence it over one’s lifetime (8). In this way, to intervene earlier in life could slow the rate of functional decline associated with aging. There is a gap in understanding early-life factors influencing late-life physical performance (8). Furthermore, there is a paucity of measures for assessing physical performance in young people as well as the fact that physical performance is rarely assessed in young individuals. Until now, tests of physical performance such as the TUG test or the 10-MWT have been used in pediatric populations with specific pathological conditions (e.g., Down Syndrome (81, 82) or neuromuscular disease (83–85)). These tests, typically employed in the geriatric population, could therefore be applied to younger populations (i.e., young adults) to evaluate physical performance despite different cut-offs may be needed.

**Table 1:** Main measures of physical performance in older people.

Measure	Description	Cut-off points
Gait speed (70, 71)	Commonly assessed through a 4-MWT. Other distances (e.g., 7 and 10 meters) have also been proposed	≤0.8 m/s
SPPB (11, 72)	Composite test including assessment of gait speed, a balance test and a chair stand test	≤8 point score
TUG (73)	The person is asked to rise from a standard chair, walk to a marker 3 m away, turn around, walk back and sit down again	≥20 s
400-MWT (74)	The person is asked to complete 20 laps of 20 m, each lap as fast as possible with up to 2 rest stops during the test	Non-completion or ≥6 min for completion
6-MWT (75)	The test is self-paced, with standardized instructions and encouragement being given as patients walk as far as possible over 6 min through a flat corridor	<400 m

4-MWT, 4 meter walk test; SPPB, short physical performance battery; TUG, timed up and go; 400-MWT, 400-meter walk test; 6-MWT, 6-min walk test.

### Omega-3 PUFAs and physical performance in early life (childhood and adolescence)

**Figure 4:**
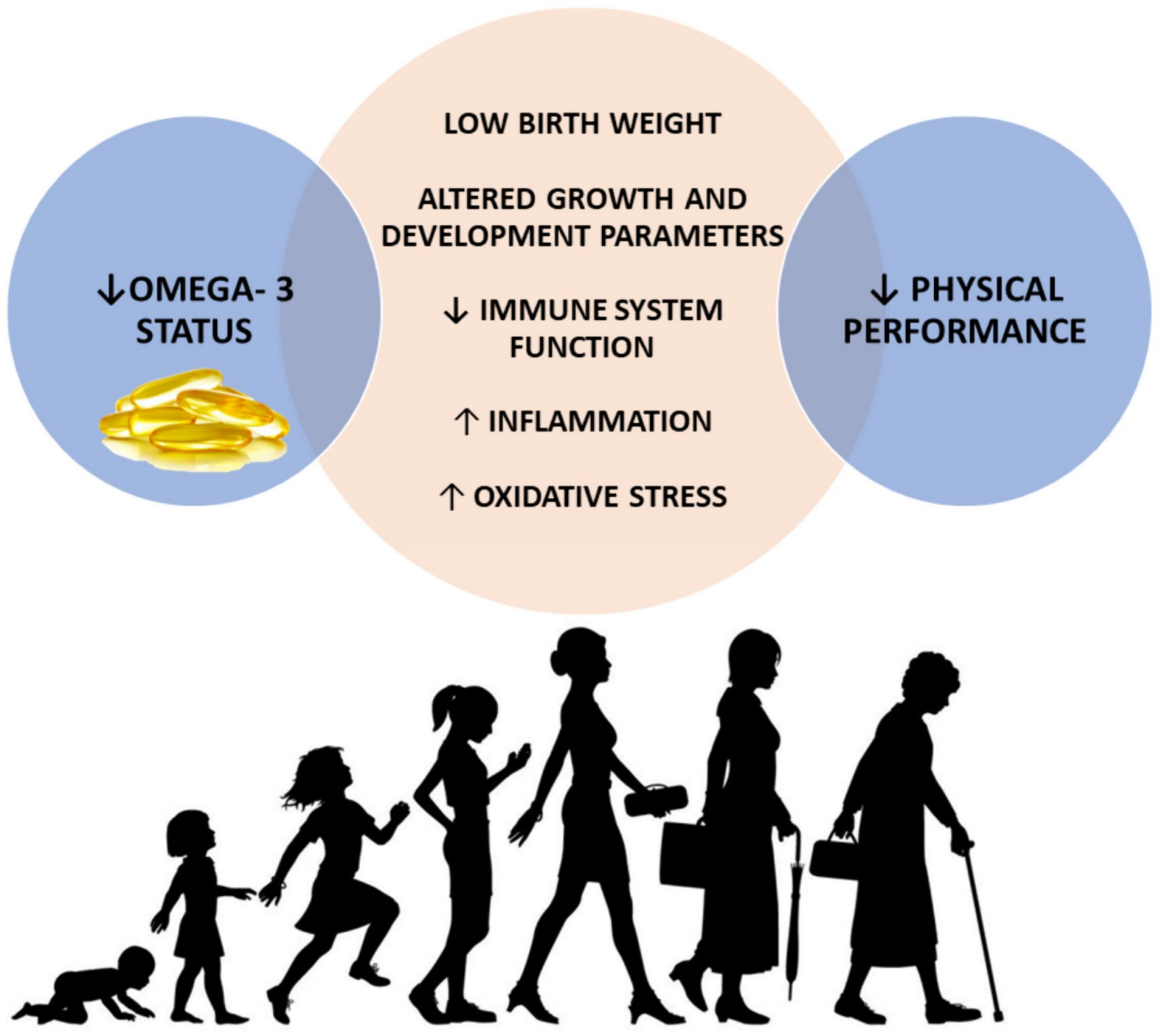
Possible mechanisms mediating the associations between low omega-3 status during early life and physical performance decline later in life. Decreased omega-3 status has been associated with low birth weight, altered growth and development parameters in early life and decreased immune function, augmented inflammation and oxidative stress across the lifespan. In turn, all these mechanisms have been largely associated with poor physical performance during older life. ↓, decreased; ↑, increased.

As the global population continues to age, there is a growing need to identify modifiable factors throughout life that influence physical function in later years. These factors may influence the peak of function achieved earlier in life determining the timing and rate of subsequent decline (Figure 1). Literature about the role of omega-3 PUFAs on physical performance in early life is scarce, although beneficial effects of these compounds on sport performance of young athletes have been reported (86, 87). Notwithstanding, some potential mechanisms by which PUFAs could exert beneficial effects on physical performance starting from early life could be argued (Figure 4).

The role of the immune system and inflammatory processes throughout the lifespan, including early and *in-utero* life, has gained growing attention as a driver of a wide spectrum of age-related chronic conditions including metabolic syndrome, type 2 diabetes, cardiovascular disease, osteoporosis and sarcopenia (88). Systemic chronic inflammation, even during childhood and pregnancy, has been reported to influence the inflammatory trajectories in later stages of life (59, 88, 89). Conversely, inflammation, defined as the elevation of pro-inflammatory cytokines (i.e., C-reactive protein, interleukin-6), has been associated with poor physical performance in both older individuals (90, 91) and young adults (i.e., sedentary young adults aged 18–35 years) (92). Inflammation could be regarded as an early determinant of the physical performance decline seen with aging, starting from childhood and even from pregnancy. Starting from pregnancy, the so-called “maternal exposome” (i.e., diet, physical activity, psychological stress and exposure to xenobiotics) influences the immune system programming of the offspring towards a more pro-inflammatory profile in adulthood (88). Poor nutrition during early life (i.e., both undernutrition and overweight and obesity) has been associated with increased levels of inflammatory markers and with consequences during adult life including cardiometabolic disease and sarcopenia (93). The omega-3 PUFAs EPA and DHA are able to partially inhibit many aspects of inflammation, including leukocyte chemotaxis, adhesion molecule expression and leukocyte-endothelial adhesive interactions, production of eicosanoids such as prostaglandins and leukotrienes from the AA, and production of pro-inflammatory cytokines (30). In this context, omega-3 supplementation during pregnancy contributes to increase omega-3 PUFAs status of the offspring (94) and influence immunological outcomes through the modification of offspring cytokine concentrations (95, 96). See et al. (97) reported for the first time that omega-3 PUFAs supplementation during pregnancy was associated with an increase in specialized pro-resolving mediators precursors in the cord blood of the offspring at birth, suggesting a beneficial role in the alleviation of low-grade inflammatory status associated with pregnancy. However, the authors found the effects were not sustained at 12 years of age highlighting that the continuation of supplementation across life course may be also a relevant factor. This is because the effects of omega-3 fatty acid consumption might require a more prolonged and continuous intervention to observe a sustained difference in pro-resolving mediators as the half-life of EPA, DHA and the resolvins is in the hour range (98, 99). The time windows (i.e., at birth and at 12 years of age), used in the study of See et al. (97), have probably been chosen because those life stages can be envisioned as critical and sensitive periods of human development, according to Barker’s ‘developmental origins of health and disease’ hypothesis (100). This theory proposes that factors that modify physiological processes during the critical developmental period in early life may exert a long-term influence on disease in adult life. In particular, early life nutritional exposures, especially during critical or sensitive periods, are significant determinants of both growth and development and later health (100). Specifically, omega-3 PUFAs supplementation during pregnancy seems to increase omega-3 PUFAs status of the newborn (94), influencing immune function by acting on cytokine concentrations (95, 96) and attenuating lipid peroxidation in the newborn (101).Various RCTs involving children and adolescents with specific pathological conditions (i.e., autism spectrum disorder, attention-deficit-hyperactivity disorder, and cystic fibrosis) have demonstrated the role of omega-3 PUFAs in countering pro-inflammatory mediators after birth (102–104).

On the other hand, low birth weight as well as altered physical growth and development parameters (at the age of 1, 7, and 12 years, respectively), have been associated with poor grip strength and physical performance in mid-to-later life (i.e., at the age of 53 and 56 years for mid-life and at the age of 65–70 years for later life) (8, 105–109). Muscle strength is a major determinant of the age-related decline of physical performance (106), as shown in Figure 1. Indeed, incorporating lifestyle modifications to maximize the peak of muscle functional capacities during early life is functional to preserve physical performance in mid-to-later life. Exploring the potential of omega-3 PUFAs to positively affect muscle strength from early life by influencing birth weight and parameters related to growth and development could be of significant interest. In this regard, a longer duration of breastfeeding has been associated with both greater grip strength in older life (110) and increased lower body explosive strength in adolescence (111). The beneficial effects of breastfeeding on grip strength could probably be mediated by breast milk fatty acids content. This is of particular interest since achieving a higher peak in muscle strength starting from early life is directly associated with a lower decline in muscle strength and, consequently, in physical performance being the latter strongly influenced by muscle strength (3, 106). Human milk contains essential dietary fatty acids such as LA and ALA, along with their metabolites AA and DHA, which play a supportive role in the growth and development of breastfed infants (112). The amount of omega-6 and omega-3 fatty acids secreted in the milk is reflective of the maternal dietary intake of PUFAs (112). The hypothesis that omega-3 PUFAs supplementation could prevent preterm birth and low birth weight has originated from studies conducted in the Faroe Islands (113). In these islands, the diet is characterized by a greater intake of marine foods compared with the population of Denmark. This likely accounts for the higher birth weights (approximately 200 g more at term) observed in babies born in this area. Furthermore, birth weights of infants from the Faroe Islands have been found to be higher than those of 33 other countries (113).

From a life course perspective, another factor to be considered is obesity (and in particular adiposity). Maternal obesity has been associated with increased fetal adiposity, especially when accompanied by gestational diabetes (93). Nutritional excess during early childhood (i.e., greater gestational weight gain, higher birth weight, faster postnatal weight gain) is associated with an increased risk of obesity, central adiposity as well as insulin resistance in adult and older life (from 20 till 70 years of age) (93, 114). Subsequently, excess weight gain during childhood and adolescence period is likely to lead to persistent overweight and obesity throughout life (115).

In this context, the role of chronic inflammation should not be overlooked since increased adiposity is characterized by the abnormal secretion of a wide range of pro-inflammatory molecules including adipokines, cytokines and chemokines thus predisposing to adult adverse conditions although inflammation is a necessary biological response to various stimuli, having defense and tissue restructuring functions (88, 116). Inflammatory trajectories across the life course could be thus probably mediated by body composition alterations as both undernutrition and overweight are associated with inflammation (93, 117, 118). In turn, obesity status, either independently or in combination with sarcopenia, has been associated with reduced physical performance in older individuals (119). Based on these considerations, omega-3 PUFAs have been suggested to play a role in the context of overweight/obesity through numerous mechanisms including the modulation of lipid metabolism and inflammation, the regulation of adipokines as well as the promotion of adipogenesis and the alteration of epigenetic mechanisms (120). A reduced red blood cell omega-3 PUFAs status has been reported in children with greater adiposity and has been associated with a suboptimal intake of omega-3 PUFAs (121). In this context, a plausible explanation may be also related to the evolution of the Western diet towards a pro-inflammatory diet rich in refined grains and ultra-processed foods and low in fruits and vegetables, thus poor in vitamins, minerals and with a suboptimal omega-3 content in favor of omega-6 PUFAs (88). However, no significant effects of omega-3 PUFAs supplementation on anthropometric parameters have been reported in children and adolescents living with overweight/obesity (122, 123).

Lower socioeconomic position (SEP) has been associated with lower serum levels of omega-3 PUFAs starting from pregnancy through adulthood (124–129). Robinson et al. (130) in a multi-cohort analysis, reported that a low SEP was independently associated with an unfavorable metabolic profile including low omega-3 status both in children (i.e., aged 7 years), adolescents (i.e., aged 15 and 17 years), adults and older adults (from 31 till 75 years of age). This is probably due to the poor quality of the diet associated with a lower socioeconomic status as reported in ethnic minorities (i.e., Latino immigrants in the U.S.), but also in urban areas of Australia (129, 131) with a reduced consumption of fruits, vegetables, whole grains, fiber, fish and seafood thus reflective of low omega-3 status (132). Additionally it should be considered that low levels and intake of omega-3 may in part be related to the limited accessibility of marine-food sources, especially in certain geographic areas (129). In a systematic review and meta-analysis, Birnie et al. (133) reported an association between lower childhood SEP and reduced physical performance in adulthood and older life (from 18 to 79 years). This connection persisted even after adjustment by adult SEP, despite the presence of heterogeneity among studies. These findings suggest that the accumulation of adverse exposures across the life course may be more predictive of the functional decline observed during aging than models considering only adult factors. It can be assumed that the association between low childhood SEP and reduced physical performance in adult and older life may be mediated, in addition to other adversities, by a poor quality of the diet, including inadequate omega-3 PUFAs intake.

It has been documented that attainment of gross motor development milestones (i.e., standing and walking) during childhood, as well as higher scores on tests measuring cognitive ability and motor coordination, are associated with enhanced physical performance in midlife, independently of other factors (8). In particular, Kuh et al. showed that the age at which an individual first walked was associated with both midlife standing balance and chair stand test, which are two out of three components of the Short Physical Performance Battery (SPPB) (8). The authors reported that better scores on cognitive ability tests at age 8 years and of motor coordination at age 15 years were associated with greater standing balance and chair standing (8). The attainment and maturation of motor and cognitive function during childhood, as well as the age-related motor and cognitive functional decline in older life, are highly integrated (134, 135), indicating that these developmental factors may be envisioned as markers of more complex cortical–subcortical neural circuits connected with higher levels of function later in life (8). This aligns with the findings of Ridler et al., who showed an anatomically related overlap between fronto-cerebellar system related to infant motor development and adult executive function (136). Similarly, Murray et al. reported that early development in the gross motor domain is associated with higher adult executive function (137). In this regard, omega-3 PUFAs are widely acknowledged to play a central role in brain function and contribute to the structure of the neuronal cell membranes (138). They are crucial for myelination and vision development during the perinatal period (139). In particular, DHA represents nearly 90% of total omega-3 PUFAs in the brain and is especially concentrated in the gray matter (140, 141). Early life accumulation of omega-3 PUFAs represents a golden opportunity for their storage in neural tissues (38, 140–142). In this context, it has been demonstrated that omega-3 PUFAs supplementation during pregnancy is associated with earlier achievement of gross motor milestones and improved cognitive development in children (143). The study by Beblo et al. (144) reported that fish oil supplementation enhanced omega-3 PUFAs levels and improved motor skills in children with phenylketonuria. Additionally, Agostoni et al. (145) in a RCT, demonstrated that infants who received DHA supplementation achieved sitting without support in a shorter period. Other studies reported contrasting results. A systematic review by U.S. Departments of Agriculture nutrition evidence reported insufficient evidence to establish a relationship between omega-3 supplementation during pregnancy and lactation with motor and visual development in infants (146). Richardson and Montgomery (147) found that in children with development coordination disorder, PUFAs supplementation (i.e., 2 capsules 3 times/day providing 558 mg of EPA, 174 mg of DHA and 60 mg of LA for 3 months) did not improve motor function, while they improved reading and spelling age and symptoms of attention-deficit/hyperactivity disorder.

In summary, the role of omega-3 PUFAs starting from early life, and even *in utero*, is intriguing given their long-lasting effects on human health through the various mechanisms discussed (e.g., birth weight, motor development, modulation of inflammation, immune response and oxidative stress) which could probably influence adult physical performance, with potential implications for the prevention of sarcopenia, frailty and disability.

### Omega-3 PUFAs and physical performance in mid to later life

Growing clinical evidence supports the positive role of omega-3 PUFAs on physical performance in mid to later life (148–150). Some studies focused on the relationship between omega-3 PUFAs supplementation and muscle strength (27, 151). As shown in Figure 1, with advancing age muscle strength tends to decline earlier and more rapidly than physical performance, especially when a lower peak in muscle strength is reached during early life. According to the latest consensus guidelines of the EWGSOP2, in the early phases of sarcopenia development, a person may result above the threshold of low physical performance despite a reduction in muscle strength (3). The reduction in muscle strength can be considered an early indicator of overt functional decline. This is evidenced by the EWGSOP2 algorithm for sarcopenia case finding where low muscle strength represents the first step in sarcopenia assessment defining probable sarcopenia and low physical performance the last step to quantify sarcopenia severity (3). Muscle strength can be thus considered as the early component, on which acting, to preserve muscle function and thus physical performance during older life. In this regard, a recent meta-analysis showed a beneficial effect of omega-3 PUFAs supplementation on lower body muscle strength while no effects were found on upper body strength (151). Bird et al. (152), in a recent meta-analysis, reported a significant relationship in favor of omega-3 PUFAs supplementation for quadriceps maximal voluntary capacity. Another meta-analysis by Rondanelli et al. (153) found no effects of omega-3 EPA plus DHA supplementation on chair rise test and handgrip strength. On the other hand, a meta-analysis of 9 RCTs showed that omega-3 PUFAs supplementation significantly increased the grip strength (154). In the Hertfordshire cohort study, Robinson et al. found a positive association between fatty fish consumption and grip strength (155). Regarding physical performance, in 2017, Frison et al. (150) reported that higher omega-3 PUFAs plasma levels were associated with lower odds of low gait speed (i.e., <0.63 m/s) in older individuals. In a cross-sectional analysis of the Multidomain Alzheimer Preventive Trial (MAPT), recruiting older adults aged 70 years and older, Fougère et al. (156) found an association between low levels of omega-3 PUFAs in red blood cell membranes and lower physical performance measured through the SPPB. However, in a secondary analysis of the MAPT trial, Rolland et al. (157) reported no significant effects of long-term omega-3 PUFAs supplementation, either alone or in combination with a multidomain lifestyle intervention comprising physical activity counseling, on the walking speed test and SPPB.

In the InCHIANTI study, including 1,273 participants between 22 and 104 years of age living in Tuscany (Italy), Abbatecola et al. (148) found that higher levels of total PUFAs, omega-3 PUFAs, and omega-6 PUFAs were associated with high physical performance (i.e., SPPB score > 9) at baseline, after adjusting for age. However, after adjusting for potential confounders, baseline 7 m walk time was associated with total PUFAs levels. Additionally, the authors found that baseline omega-3 PUFAs levels were inversely associated to the risk of developing a decline in SPPB to scores ≤9, while the omega-6/omega-3 ratio was associated with a higher risk of SPPB decline and with a longer time to walk 7 meters.

Hutchins-Wiese et al. (158), in a RCT recruiting 126 postmenopausal women, demonstrated that omega-3 PUFAs supplementation resulted in greater physical performance, measured by change in walking speed. On contrary, Krzymińska-Siemaszko et al. (159) did not observe an improvement in physical performance after 12 weeks of omega-3 PUFAs supplementation in a sample of older people with low muscle mass. A recent meta-analysis of RCTs in older adults (153), reported that daily omega-3 PUFAs supplementation is associated with a reduction in the time of the TUG test, while no statistically significant effect was found on 4-MWT. These results are in line with another recent meta-analysis (151), focused on people aged 55 years and older, which reported a significant association between omega-3 supplementation and lower TUG but not on walking speed. However, in another systematic review and meta-analysis, it was found that there were minor benefits of omega-3 supplementation for TUG performance, while subgroup analyses showed that omega-3 PUFAs supplements at more than 2 g/day may contribute at improving walking speed, especially if the intervention is carried out for more than 6 months (160).

In summary, evidence from interventional studies and meta-analyses gave controversial results on the beneficial effect of omega-3 PUFAs on physical performance parameters. As outlined by some authors, small study size and heterogeneity in the intervention protocol, including the ratios between EPA and DHA, may have limited the applicability of these results.

## Conclusion

Mechanisms through which omega-3 PUFAs could benefit muscle function including physical performance parameters across the life course are intriguing. However, it should be considered that few interventional studies explored the effects of omega-3 PUFAs supplementation on physical performance, with varying duration, dosage and use (i.e., alone or in combination with other interventions). This has led to controversial results reported in literature. However, it seems that omega-3 PUFAs supplementation at high doses (i.e., more than 2 g/day) and for longer periods (i.e., for more than 6 months) may contribute to improving physical performance (e.g., walking speed) in older people. Larger and high-quality RCTs are needed to fully elucidate the beneficial effects of omega-3 PUFAs supplementation on muscle function parameters. Although, a direct association in early life is not available in literature, some mechanisms by which omega-3 PUFAs may contribute to improved physical performance could be hypothesized. The integration of physical function measures in future RCTs would be of great interest to explore whether omega-3 PUFAs could contribute to improved muscle function parameters, starting from early life and extending throughout the lifespan.

## Author contributions

DA: Writing – original draft, Writing – review & editing. CB: Writing – original draft, Writing – review & editing. VC: Writing – review & editing. GS: Writing – review & editing. CA: Writing – review & editing. TL: Writing – review & editing. AM: Writing – review & editing.
